# An update on drug-drug interactions for care of the acutely ill in the era of COVID-19

**DOI:** 10.1093/ajhp/zxad152

**Published:** 2023-06-27

**Authors:** Asad E Patanwala, Nynke G L Jager, John J Radosevich, Roger Brüggemann

**Affiliations:** Faculty of Medicine and Health, School of Pharmacy, The University of Sydney, Camperdown, New South Wales, and Department of Pharmacy, Royal Prince Alfred Hospital, Camperdown, New South Wales, Australia; Department of Pharmacy, Radboud University Medical Center, Nijmegen, and Radboudumc Institute for Health Sciences, Radboud University Medical Center, Nijmegen, the Netherlands; Department of Pharmacy Services, Dignity Health–St. Joseph’s Hospital & Medical Center, Phoenix, AZ, USA; Department of Pharmacy, Radboud University Medical Center, Nijmegen, and Radboudumc Institute for Health Sciences Center of Expertise in Mycology Radboudumc/CWZ, Radboud University Medical Center, Nijmegen, the Netherlands

**Keywords:** critical care, cytochrome P-450 enzyme system, drug interactions, membrane transport proteins, patient safety, pharmacokinetics

## Abstract

**Purpose:**

To provide key pharmacological concepts underlying drug-drug interactions (DDIs), a decision-making framework, and a list of DDIs that should be considered in the context of contemporary acutely ill patients with COVID-19.

**Summary:**

DDIs are frequently encountered in the acutely ill. The implications of DDIs include either increased risk of drug toxicity or decreased effectiveness, which may have severe consequences in the acutely ill due to lower physiological and neurocognitive reserves in these patients. In addition, an array of additional therapies and drug classes have been used for COVID-19 that were not typically used in the acute care setting. In this update on DDIs in the acutely ill, we provide key pharmacological concepts underlying DDIs, including a discussion of the gastric environment, the cytochrome P-450 (CYP) isozyme system, transporters, and pharmacodynamics in relation to DDIs. We also provide a decision-making framework that elucidates the identification of DDIs, risk assessment, selection of alternative therapies, and monitoring. Finally, important DDIs pertaining to contemporary acute care clinical practice related to COVID-19 are discussed.

**Conclusion:**

Interpreting and managing DDIs should follow a pharmacologically based approach and a systematic decision-making process to optimize patient outcomes.

**KEY POINTS**

- Previously uncommon drug classes used in the acutely ill have introduced new drug-drug interactions (DDIs) in patients with COVID-19.
- Pharmacological concepts to consider include the gastric environment, cytochrome P-450 isoenzyme system, transporters, and pharmacodynamics in relation to DDIs.
- The decision-making for DDIs is complex in the acutely ill and should include a decision-making framework.

Drug-drug interactions (DDIs) are frequently encountered in the acutely ill. Among the most severely ill patients, such as those in the intensive care unit (ICU), 58% encounter at least one potential DDI, with some studies identifying up to 5 potential DDIs per patient.^1-3^ Most DDIs in the literature are considered to be potential unless a clinically relevant outcome occurs.^1^ For the purpose of this article, we have referred to these simply as DDIs. More recently, introduction of new therapies for the management of coronavirus disease 2019 (COVID-19) has had implications for practice due to DDIs that were not previously commonly encountered in acutely ill patients. The implications of a DDI include either increased risk of drug toxicity or decreased effectiveness. This may have severe consequences in more severely ill patients with COVID-19 due to the lower physiological and neurocognitive reserves of such patients.^4^ In a recent systematic review of patients with COVID-19, numerous DDIs were associated with harm, most of which would have been preventable if identified with drug interaction tools.^5^ Thus, an updated review of DDIs to guide clinicians who manage acutely ill patients with COVID-19 is needed.

In this clinical consultation, we provide key pharmacological concepts underlying DDIs, a decision-making framework for clinicians, and a list of DDIs that should be considered in the context of contemporary acutely ill patients with COVID-19.

## Decision-making framework

**Figure 1.**
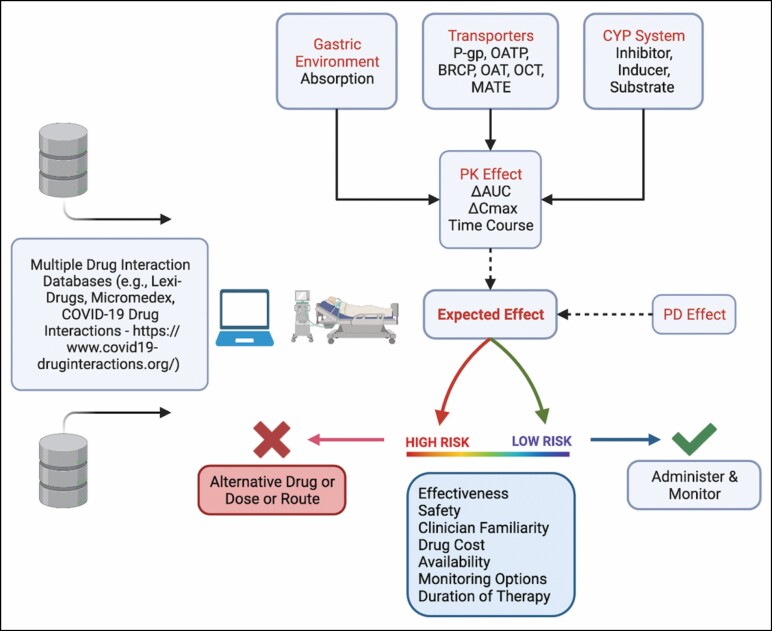
Flow diagram of the decision-making framework. AUC indicates area under the curve; BRCP, breast cancer resistance protein; *C*_max_, maximum concentration; CYP, cytochrome P-450; MATE, multidrug and toxin extrusion; OAT, organic anion transporter; OATP, anion-transporting polypeptides; OCT, organic cation transporter; PD, pharmacodynamic; P-go, P-glycoprotein; PK, pharmacokinetic.

The decision-making framework for DDIs in patients with COVID-19 (Figure 1) is conceptually similar to that for DDIs in general. The starting point is identification of a DDI. One approach is to routinely screen patients’ medication lists and newly prescribed therapies using a few different compendia that can identify DDIs. More than one compendium (eg, Micromedex and Lexi-Drugs) is preferred, as there is not exact agreement between sources.^6^ The COVID-19 drug interactions webpage is another resource to help clinicians navigate DDIs with emerging COVID-19 therapies.^7^ Identification of a DDI is followed by an assessment of changes in expected area under the curve (AUC) and/or maximum concentration, the time course of these changes, and pharmacodynamic (PD) effects. These concepts are further elaborated in the next section. Finally, a determination of risk is needed for either continuation of the interacting drugs with monitoring or use of alternative therapy.

Circumstances for severely ill patients such as those in the ICU may allow for monitoring and treatment of adverse effects to mitigate potential harm from DDIs. Monitoring of serum drug concentrations, physical status, or biomarkers indicative of harm may allow for the use of a drug-drug combination even when an interaction is present.^8^ For example, continuation of a statin may be acceptable if serum creatinine kinase levels and renal function are monitored. Similarly, QTc-prolonging agents may be continued in patients with a shorter baseline QTc interval and ongoing electrocardiogram monitoring. In an analysis of management of potential DDIs in the ICU, monitoring was the advised management strategy in 81% of cases.^8^ Thus, it is common to continue certain therapies with DDIs in the ICU, more so than in other environments. A decision to continue drugs in the presence of DDIs requires consideration of the expected effects of the DDI, risk evaluation, the availability of alternative therapies, monitoring, and the harm mitigation strategies available.^9^

## Key pharmacological concepts

Key pharmacological concepts in the acutely ill related to COVID-19 DDIs pertain to the gastric environment, the cytochrome P-450 (CYP) isoenzyme system, transporters, and PD effects. These are briefly discussed below and are depicted in Figure 1. Standard terminology for DDIs refers to drugs in a DDI pair as either the “perpetrator” or “victim,” or in some cases both.^10^ The perpetrator (inducer or inhibitor) affects the pharmacokinetic (PK) and PD behavior of the victim (substrate).

### Gastric environment

Severely ill patients such as those in the ICU may have poor gastrointestinal perfusion, changes in gastric pH, and altered gastrointestinal motility.^11^ This can result in highly variable and often reduced bioavailability of orally or enterally administered COVID-19 therapies.^11^ One known DDI relevant to COVID-19 therapies that pertains to the gastric environment involves the combination of oral magnesium antacids and corticosteroids. The absorption of oral dexamethasone was evaluated via measurement of urinary excretion of 11-hydroxycorticosteroids in 6 healthy individuals.^12^ This analysis showed a reduction of approximately 75% in the bioavailability of dexamethasone when it was administered concurrently with magnesium trisilicate. The probable mechanism involves adsorption of dexamethasone on the surface of the antacid, and this interaction becomes relevant when oral or enteral corticosteroids are used. In these circumstances, the dose of dexamethasone should be separated from that for the antacid by more than 2 hours.

### CYP isoenzyme system

Most DDIs with COVID-19 therapies involve the CYP isoenzyme system. A detailed description of the CYP isoenzyme system and its role in drug variability has previously been published.^13^ Perpetrator drugs can be either an inhibitor or an inducer of CYP enzymes. An inhibitor slows down the metabolism of the victim drug, while an inducer increases its metabolism. The Food and Drug Administration (FDA) maintains a useful resource for clinicians that describes substrates, inhibitors, and inducers of CYP enzymes.^14^ When evaluating DDIs in acutely ill patients with COVID-19, the extent of the change in exposure (ie, the change in the AUC of the victim drug) and the time course of this change are important considerations. The strength of the inhibitor (ie, perpetrator) and the sensitivity of the substrate (ie, victim) both determine the extent of change in exposure. Strong, moderate, and weak inhibitors increase the AUC of sensitive substrate by ≥5-fold, ≥2-fold to <5-fold, and ≥1.25-fold to <2-fold, respectively.^14^ For example, ritonavir (used with nirmatrelvir for COVID-19) is a strong inhibitor of CYP3A4. Thus, it can increase the AUC of some victim drugs by 5-fold or more, which can have severe consequences. The time course of enzyme inhibition is also important to consider. Onset of inhibition with ritonavir occurs upon first administration, while extinction of inhibition is related to the half-life of ritonavir and the time required for the activity of the CYP enzyme to return.^15^ This means that a DDI should be expected for several days, even when nirmatrelvir-boosted ritonavir is discontinued upon hospital admission.

DDIs may also be genotype dependent. For instance, voriconazole is predominantly metabolized by CYP2C19 and to a lesser extent by CYP3A4 and CYP2C9.^16^ If there is decreased function of CYP2C19, metabolism by CYP3A4 becomes the dominant pathway. This was highlighted by a DDI study of voriconazole and ritonavir (a strong CYP3A4 inhibitor) in healthy individuals.^17^ In this study, the voriconazole AUC increased 1.5-fold in ultrarapid metabolizers and 9.1-fold in poor metabolizers. In the severely ill, the CYP isoenzyme system and enzyme expression are subject to a substantial degree of variability that is further affected by the presence of inflammation. Evidence has indicated that increased inflammatory status results in isoform-specific changes to the metabolic activity of CYP enzymes.^18^ The activity of some CYP enzymes increases while that of others decreases in such patients. For example, in patients with moderate to severe COVID-19, the activity of CYP2C19 and CYP3A4 decreased by 75% and 23%, respectively, whereas the activity of CYP2C9 increased by 56%.^19^ The extent of the change in the AUC of a victim drug in the presence of inflammation and a DDI can be difficult to predict.

### Transporters

The human body has hundreds of transporters, but clinical relevance is currently known for only a minority of these.^20^ FDA has provided guidance that clinical DDI studies should be conducted for new drugs that are substrates of the following types of transporters: P-glycoprotein (P-gp), anion-transporting polypeptide (OATP), breast cancer resistance protein (BCRP), organic anion transporter (OAT), organic cation transporter, and multidrug and toxin extrusion (MATE) proteins.^21^ These transporters can play an important part in DDIs because they regulate the access of a substrate to metabolizing enzymes, control the influx and efflux of drugs from enterocytes and hepatocytes, or influence renal secretion. Examples of inducers, inhibitors, and substrates for these transporters are listed by FDA.^14^ The OAT and P-gp transporters are most pertinent with regard to DDIs involving COVID-19 therapies and are discussed later in this article in regard to baricitinib, ritonavir-boosted nirmatrelvir, and remdesivir.

### PD effects

PD interactions are characterized by the ability of a DDI pair to have an altered combined effect that is not related to a change in drug concentration.^22^ These PD interactions are typically additive but can also be synergistic (where the combined effect is more than the sum of the individual effects) and in some cases antagonistic (diminished or neutral effect). Examples of PD DDIs in COVID-19 relate to the use of multiple medications such as interleukin-6 (IL-6) inhibitors, Janus kinase (JAK) inhibitors, and corticosteroids that can all cause immunosuppression. Generally, PD DDIs will result in increased toxicity rather than decreased effectiveness.

## COVID-19 drug interactions

Targeted therapies for COVID-19 have challenged acute care providers with new DDIs due to the use of some drug classes that were previously not routinely used in the hospital setting. As a starting point, therapies for COVID-19 currently recommended by the National Institutes of Health (NIH)^23^ will be discussed as these are most likely to be seen in contemporary clinical practice. We also acknowledge that recommendations can change and new therapies are being investigated. The mechanisms for DDIs for each COVID-19 drug and the implications are shown in Table 1. While it is not practical to list all possible DDIs, their consequences, and recommendations, we have provided examples that highlight the types of issues faced in the acutely ill.

**Table 1.** Drug-Drug Interactions With COVID-19 Therapies

Drug	Mechanism of interaction^a^	Comments
**Monoclonal antibodies**		
Bebtelovimab	Not a substrate, inhibitor, or inducer of CYP enzymes or transporters	No clinically relevant interactions
Tixagevimab/cilgavimab	Not a substrate, inhibitor, or inducer of CYP enzymes or transporters	No clinically relevant interactions
**Corticosteroids**		
Dexamethasone	• CYP3A4 substrate (major) and inducer of CYP3A4 (moderate) • Immunosuppression • Adsorbed on surface of magnesium antacids • Decreased potassium concentration	• Increase concentration with strong CYP3A4 inhibitors; monitoring vs dose reduction • May decrease concentration of CYP3A4 substrates • Increased infection risk with other drugs that suppress the immune system • Separate by 2 hours from magnesium antacids when oral route used • Monitor potassium with other drugs that decrease potassium (eg, diuretics)
**IL-6 inhibitors**		
Tocilizumab	Immunosuppression	Increased infection risk with other drugs that suppress the immune system
Sarilumab	Immunosuppression	Increased infection risk with other drugs that suppress the immune system
**JAK inhibitors**		
Baricitinib	• Substrate of strong OAT3 transporter inhibitors • Immunosuppression	• AUC increased 2-fold with strong OAT3 transporter inhibitors (eg, probenecid, teriflunomide); decrease dose to half • Increased infection risk with other drugs that suppress the immune system
Tofacitinib	• Substrate of CYP3A4 (major) and CYP2C19 (minor) • Immune suppression	• Decrease dose to half with strong CYP3A4 inhibitors (eg, ketoconazole) or a moderate CYP3A4 inhibitor + strong CYP2C19 inhibitor (eg, fluconazole) • Increased infection risk with other drugs that suppress the immune system
**Antivirals**		
Ritonavir-boosted nirmatrelvir	Inhibits CYP3A4 (strong) and CYP2D6 (weak)	• Contraindication or precaution with major CYP3A4 substrates • DDI present for 3-7 days after cessation
Molnupiravir	Not a substrate, inhibitor, or inducer of CYP enzymes or transporters	No clinically relevant interactions
Remdesivir	• Minor substrate for several CYP enzymes or transporters • Possibly prolongs QTc interval • Decreased antiviral effect with hydroxychloroquine or chloroquine	• PK DDIs unlikely • Increased QTc possible with other drugs that prolong QTc • Avoid hydroxychloroquine or chloroquine

Abbreviations: AUC, area under the curve; CYP, cytochrome P-450; DDI, drug-drug interaction; IL-6, interleukin-6; JAK, Janus kinase; PK, pharmacokinetic.

^a^Inhibitors and inducers categorized as weak, moderate, or strong; substrates categorized as minor or major.

### Monoclonal antibodies

Monoclonal antibodies target spike protein components of severe acute respiratory syndrome coronavirus 2 (SARS-CoV-2). These drugs include bebtelovimab and the combination product tixagevimab/cilgavimab, which have a molecular weight of approximately 144 kDa and 149 kDa, respectively.^24,25^ Biologics with a molecular weight of more than 69 kDa are not renally eliminated and do not require PK analysis in the context of renal impairment by FDA.^24,25^ In addition, these monoclonal antibodies are not metabolized by CYP enzymes. As these agents are indicated in the outpatient setting for those at high risk of progression to severe disease, it is possible that these agents have been administered to some patients before hospital admission. However, there are no known DDIs with these drugs, and their prior use would not affect selection of therapies during hospitalization.

### Corticosteroids

The best evidence for use of corticosteroids to treat COVID-19 comes from dexamethasone.^26^ In the RECOVERY trial, a dose of 6 mg was administered intravenously (IV) or orally daily for 10 days.^26^ However, hydrocortisone was also shown to be beneficial in a severely ill cohort in the REMAP-CAP trial, with a dose of 50 mg IV every 6 hours or 100 mg IV every 8 hours, suggesting a class effect.^27^ The equivalent corticosteroid dose based on glucocorticoid effects was higher in REMAP-CAP than in the RECOVERY trial (200-300 vs 160 mg hydrocortisone equivalents per day). This has led to interest in using higher doses of dexamethasone (12-20 mg daily).^28^ An ongoing investigation from the RECOVERY trial using higher doses of dexamethasone in the first 5 days (20 vs 6 mg daily) has shown an increased risk of mortality in the high-dose group, with contributing factors of hyperglycemia, non–COVID-19 infection, and gastrointestinal bleeding.^29^ Although definitive evidence of the optimal corticosteroid regimen in COVID-19 is emerging, we can presume that DDIs that can increase the dexamethasone AUC by more than 3-fold relative to the standard regimen of 6 mg daily could be harmful. Use of corticosteroids in hospitalized patients, especially in the critically ill, is not new. However, it is important not to overlook the fact that dexamethasone is a CYP3A4 substrate (major) and an inducer of CYP3A4 (weak). For example, itraconazole, which is a strong inhibitor of CYP3A4, has been shown to increase the dexamethasone AUC by 3.3- to 3.7-fold.^30^ It is possible that other strong CYP3A4 inhibitors may cause similar increases in dexamethasone exposure. We suggest that, when a standard dose is used in the presence of a strong CYP3A4 inhibitor, there should be strict monitoring (eg, at least every 6 hours) and correction of blood glucose levels (eg, to less than 180 mg/dL). A temporary dose reduction to 4 mg per day in the presence of a strong inhibitor may be considered in rare circumstances (eg, in patients who are not severely ill and have difficult-to-control blood glucose levels), but this requires a careful risk-benefit assessment for a given patient to ensure that such a dose reduction does not impede their recovery from COVID-19.

Dexamethasone is a mild inducer of CYP3A4, which is unlikely to have important clinical implications. However, drug concentrations for CYP3A4 substrates with a narrow therapeutic index (eg, tacrolimus and cyclosporin) should be monitored at least once during hospitalization after dexamethasone initiation.^31^ Corticosteroids are also known to enhance potassium elimination. Patients using diuretics should have their potassium concentration monitored daily during hospitalization as part of their metabolic panels.^32^ A low potassium level can be harmful particularly in patients with cardiac dysrhythmias or those managed with digoxin. Finally, dosing for magnesium antacids should be separated by at least 2 hours from that for corticosteroids if an oral corticosteroid is used, as adsorption can decrease the AUC of the corticosteroid by more than 75%.^12^

### IL-6 inhibitors

The IL-6 inhibitors investigated for COVID-19 include tocilizumab and sarilumab,^33,34^ which have been administered IV as single doses of 8 mg/kg (up to 800 mg) and 400 mg, respectively. In the RECOVERY trial, 29% of patients were administered a second dose of tocilizumab based on clinician discretion.^33^ While these agents are FDA approved for chronic use in rheumatoid arthritis, clinicians in the acute care setting may be more familiar with tocilizumab, which is used for cytokine release syndrome at doses similarly high to those used for COVID-19. The terminal half-life of tocilizumab is 21 days,^35^ and, from a DDI perspective, it is expected that the effects of single doses used for COVID-19 would last at least for the duration of hospitalization. Blockade of IL-6 helps normalize previously upregulated CYP enzyme activity due to chronic inflammation,^35^ and DDIs involving CYP substrates and IL-6 inhibitors may therefore be flagged by databases in the context of chronic inflammatory states such as rheumatoid arthritis. However, this is not relevant to COVID-19 as any change in CYP activity due to treatment would just return the patient to their baseline state. Similar to other monoclonal antibodies, these drugs have a high molecular weight and are not eliminated renally.^35^ Additionally, they are not eliminated via hepatic pathways but should be used with caution or avoided in patients with elevated levels of liver enzymes (alanine transaminase levels more than 5 times the upper limit of normal) due to their potential to cause liver injury.^36^ The mechanism for this is unknown but may be related to IL-6 inhibition or effects on the immune system. No DDIs are expected due to renal changes or CYP enzymes.

IL-6 inhibitors have immunosuppressive and hematological effects. Caution is advised in patients who are already immunosuppressed as a consequence of other therapies, with an absolute neutrophil count of less than 500 cells/µL or a platelet count of less than 50 × 10^3^ cells/µL.^36^ For example, one expected interaction is between corticosteroids and IL-6 inhibitors, as these are both indicated in severely ill patients with COVID-19 requiring oxygen or ventilatory support.^23^ This is a PD DDI that would likely be flagged in databases. However, even though there is increased risk for superinfection, dexamethasone in combination with either an IL-6 inhibitor or JAK inhibitor has been well tolerated and led to improved outcomes in large trials.^33,34^ Thus, our decision-making framework suggests that this combination is appropriate.

### JAK inhibitors

Bariticinib and tofacitinib are the JAK inhibitors with the most evidence for use based on COVID-19 clinical trials.^37,38^ These are administered orally or enterally for hospitalized patients and were used in the aforementioned trials for 10 to 14 days. It is important to recognize that these agents have different interaction profiles. Baricitinib is a CYP3A4 substrate; however, studies in healthy individuals have shown that strong inhibitors (eg, ketoconazole) or inducers (eg, rifampin) do not result in a meaningful change in baricitinib AUC.^39^ Baricitinib is also a substrate for several transporters, including OAT3, P-gp, BCRP, and MATE2-K. However, only changes to OAT3 activity have been shown to result in an important change in its elimination, with the OAT3 inhibitor probenecid increasing the AUC of baricitinib by 2-fold.^39^ FDA has dose reduction recommendations (ie, reduce the dose to half) for baricitinib when it is used with strong transporter (OAT3) inhibitors.^39^ Another strong OAT3 inhibitor is teriflunomide, which is used for multiple sclerosis. Fortunately, use of strong OAT3 inhibitors is uncommon.

Tofacitinib is a substrate for CYP3A4 (major) and CYP2C19 (minor). Administration of a strong inhibitor of CYP3A4 (ketoconazole) increased the AUC by 103%, and a strong CYP3A4 inducer reduced the AUC by 84%.^40^ Fluconazole is a moderate CYP3A4 inhibitor and a strong CYP2C19 inhibitor. Co-administration of fluconazole increased the AUC of tofacitinib by 79%.^40^ FDA has a dose reduction recommendation (ie, reduce the dose from 5 mg twice daily to 5 mg once daily) for tofacitinib when it is used with a strong CYP3A4 inhibitor or the combination of a moderate CYP3A4 inhibitor and a strong CYP2C19 inhibitor.^41^ Numerous drugs inhibit these CYP enzymes, requiring regular checking for interactions via a database based on a patient’s medication profile, with subsequent reduction in the dose of tofacitinib if indicated.

JAK inhibitors also have immunosuppressive effects. Thus, there is a PD interaction when using them with dexamethasone. However, similar to the IL-6 and dexamethasone combination, use of a JAK inhibitor and dexamethasone is appropriate according to our decision-making framework. Clinical trials have shown beneficial effects when these medications are used in combination for COVID-19, although a risk of superinfections is present.^37,38,42^ Use of JAK inhibitors with IL-6 inhibitors is emerging in small case series of patients with COVID-19, with no evidence of serious harm,^43^ but this combination has not been thoroughly investigated and cannot be recommended by our decision-making framework due to an increased risk of secondary infections and lack of evidence.^39,41^

### Antivirals

 NIH guidelines recommend ritonavir-boosted nirmatrelvir for nonhospitalized patients with COVID-19 who are at high risk of progressing to severe disease.^23^ Ritonavir, a well-known perpetrator, is a P-gp inhibitor and a strong CYP3A4 inhibitor. A DDI in this context is built into the formulation by the incorporation of ritonavir, resulting in a boosted effect of nirmatrelvir. In the case of nirmatrelvir, drug exposure is significantly higher when this is co-administered with ritonavir due to CYP3A4 inhibition, enhancing the effect against COVID-19.

When patients treated with ritonavir-boosted nirmatrelvir in the community are admitted to the ICU, providers must decide whether to continue this therapy. In some circumstances, hospital admission may be related to a DDI involving ritonavir-boosted nirmatrelvir. If the admission is due to worsening COVID-19 associated with oxygen/ventilatory support, ritonavir-boosted nirmatrelvir is likely to be discontinued. Although transition to dexamethasone plus an IL-6 or JAK inhibitor is warranted, this does not prevent the occurrence of DDIs due to ongoing CYP inhibition.^23^ If the admission is unrelated to COVID-19, completion of the ritonavir-boosted nirmatrelvir course would be reasonable. In either scenario, DDIs are likely to be apparent as inhibition of CYP3A4 can be sustained for 3 to 7 days following discontinuation, with the delay mostly due to the time required for liver enzyme activity to return to normal after suppression.^44-46^ Furthermore, ritonavir can induce other CYP enzymes (CYP1A2 [weak], CYP2B6 [moderate], CYP2C19 [weak], and CYP2C9 [weak]), with the effect potentially lasting for a few weeks. This lagging effect on CYP enzymes increases the probability of unrecognized DDIs because discontinued therapies do not appear on the patient’s active medication profile. There are numerous drugs that are major substrates for CYP3A4 and P-gp; while it is impractical for us to provide a list of these medications that may be used in the hospital and their associated risks, it is important to recognize that sensitive substrates may have a 5-fold increase or greater in AUC. In most circumstances, we would recommend withholding the substrate temporarily if clinically possible and/or using an alternative agent. For example, the AUC of simvastatin increased 31-fold in the presence of ritonavir.^47^ The risk for rhabdomyolysis with such an increase in AUC is high, and withholding the statin in this situation is clearly required.

The other oral antiviral used for COVID-19 is molnupiravir. It is not a substrate for CYP enzymes or common human drug transporters,^48^ and there are therefore no known drug interactions with this agent. While this is appealing from a DDI perspective, use of molnupiravir is no longer favored relative to ritonavir-boosted nirmatrelvir due to the results of the PANOMAMIC trial, which did not show a reduction in the frequency of hospitalization or death with molnupiravir.^49^

Remdesivir is an IV administered antiviral and one of the first agents to show a benefit by reducing the time to clinical recovery in COVID-19.^50^ Remdesivir is a minor substrate for several metabolic and transport pathways, including plasma and tissue esterases, CYP enzymes (CYP3A4, CYP2D6, and CYP2C8), and transporters (OATP1B1 and P-gp). DDI studies with remdesivir are lacking. Thus, the potential for interactions is theoretical and no dose adjustments are recommended. Although hydroxychloroquine and chloroquine are not recommended and are potentially harmful in COVID-19, these agents may also antagonize the antiviral effect of remdesivir.^51^ A published case report highlights that remdesivir’s P-gp–mediated efflux from hepatocytes may be impaired by P-gp inhibitors, potentially contributing to an enhanced hepatotoxic effect of remdesivir.^52^ There is also a possible risk for increased QTc interval with remdesivir.^53^ Thus, QTc interval monitoring is recommended when there is a DDI with another agent that can prolong QTc.

## Conclusion

When new drugs are introduced for clinical use, such as those for COVID-19, the new drug application is supplemented with preclinical information that is submitted to regulatory authorities. This provides the basic information required to assess the potential for DDIs based on affinity for metabolizing enzymes and transporters, which we have utilized in this article. In addition, many of the therapies recommended for COVID-19 were repurposed from other indications, which has allowed for a body of postmarketing studies that provides additional information to guide DDI decisions. Decisions can be guided by the extent of expected change in PK and PD parameters, anticipated clinical effects, and a risk evaluation. Among the various COVID-19 therapies, ritonavir-boosted nirmatrelvir has been one of the most challenging for clinicians from a DDI perspective. Clinicians in the acute care environment should carefully consider that, even after ritonavir-boosted nirmatrelvir is ceased upon hospitalization, clinically relevant DDIs may be present. A systematic and pharmacologically based decision-making process should be applied to improve outcomes and avoid harm.

## Data Availability

No new data were generated or analyzed in support of this article.
